# Supplementary material for: Intraoperative complications of cataract surgery using a low-energy femtosecond laser: Results from a real-world high-volume setting
Source: PLoS One. 2022 Dec 15;17(12):e0279023. doi: 10.1371/journal.pone.0279023 (PMC9754167; doi:10.1371/journal.pone.0279023)
Supplement: Attachment — Submitted filename: Response to Reviewers.pdf [file pone.0279023.s003.pdf]

Dear Reviewer

thank you very much for your effort and review of this paper. Please find enclosed our revised manuscript titled “Intraoperative complications of cataract surgery using a low-energy femtosecond laser: Results from a real-world high-volume setting”.

Following the comments of Reviewer #1, we arranged the references in the manuscript in the systematic fashion. The reference numbers are now in the right order.

We look forward to hearing from you at your earliest convenience.

Sincerely,

Julia Riemey

Research Associate

Dardenne Eye Hospital, Friedrich-Ebert-Str. 23-25, 53177 Bonn, Germany.

Tel.: +49 228 8303-118

Fax: +49 228 8303-129

E-Mail: julia.ri@gmx.de
