# Supplementary material for: Intraoperative complications of cataract surgery using a low-energy femtosecond laser: Results from a real-world high-volume setting
Source: PLoS One. 2022 Dec 15;17(12):e0279023. doi: 10.1371/journal.pone.0279023 (PMC9754167; doi:10.1371/journal.pone.0279023)
Supplement: Attachment — Submitted filename: Response to Reviewers.docx [file pone.0279023.s002.docx]

Dear Reviewer

thank you very much for your effort and review of this paper. Please find enclosed our revised manuscript titled “Intraoperative complications of cataract surgery using a low-energy femtosecond laser: Results from a real-world high-volume setting”.

Following the suggestions of Reviewer #2, we have developed and expanded the concept of cataract surgery using low-energy lasers. The low-energy-concept is now carried out in detail.

Following the comments of Reviewer #1, we carefully reworked our abbreviations used in the manuscript. Mentioned abbreviations are now explained with their first use.

The revised manuscript has subsequently been carefully reviewed by an experienced editor whose first language is English and who specializes in editing papers written by scientists whose native language is not English.

We look forward to hearing from you at your earliest convenience.

Sincerely,

**Julia Riemey**

Research Associate

Dardenne Eye Hospital, Friedrich-Ebert-Str. 23-25, 53177 Bonn, Germany.

Tel.: +49 228 8303-118

Fax: +49 228 8303-129

E-Mail: [forschung@dardenne.de](mailto:forschung@dardenne.de)
